# Rift Valley Fever in Goats, Cameroon

**DOI:** 10.3201/eid1204.051428

**Published:** 2006-04

**Authors:** Matthew LeBreton, Sally Umlauf, Cyrille F. Djoko, Peter Daszak, Donald S. Burke, Paul Yemgai Kwenkam, Nathan D. Wolfe

**Affiliations:** * Walter Reed Johns Hopkins Cameroon Program, Yaoundé, Cameroon;; † Tufts University School of Veterinary Medicine, North Grafton, Massachusetts, USA;; ‡ University of Yaoundé, Yaoundé, Cameroon;; § Consortium for Conservation Medicine, New York, New York, USA;; ¶ Johns Hopkins University, Baltimore, Maryland, USA;; # Ministry of Livestock, Fisheries and Animal Industries, Yaoundé, Cameroon

**Keywords:** Zoonoses, Cameroon, Goats, Rift Valley fever virus, letter

**To the Editor:** Rift Valley fever (RVF) is caused by an RNA virus (*Phlebovirus*, *Bunyaviridae*), which is carried by mosquito vectors (*1*). In nature, it is only known in Africa and the Arabian Peninsula. In the central African region, RVF has been reported in humans and livestock in the savanna of northern Cameroon and Chad (*2**,**3*) and from forests in the Central African Republic (*4*). Human epidemics are sometimes preceded by an increase in RVF virus (RVFV) prevalence in domestic ruminants, which manifests as increased abortions and high neonatal deaths (*3*). Human epidemics can have serious health implications, as demonstrated by the most recent outbreaks in Kenya in 1997 and 1998 (*5*) and Saudi Arabia in 2001 (*6*).

In June 2003, 14 goats from an urban market in Yaoundé (3.9°N, 11.5°E), Cameroon, and 36 goats from 3 adjacent villages in tropical lowland rainforest ≈80 km south of Mvangan (2.4°N, 11.8°E), Cameroon, were sampled. The goats in the rural villages were bred locally and allowed to roam freely throughout the villages. No vaccinations had been given to goats in the rural sites. Goats in the urban market in Yaoundé generally originated in northern Cameroon and had been transported by train to Yaoundé. Owners did not report high levels of abortion or high neonatal deaths.

Jugular blood was collected in a 5-mL dry Vacutainer tube and centrifuged within 48 hours of collection. The frozen sera were shipped on ice to the Onderstepoort Veterinary Institute, the United Nations Food and Agriculture Organization reference laboratory for RVFV. An indirect enzyme-linked immunosorbent assay (I-ELISA) was used to detect RVFV immunoglobulin G (IgG) antibodies in 26 samples. In this assay, optical density readings were converted to a percentage of high-positive control serum (PP) and positive samples were those with PP >10. These samples were further tested with RVFV hemagglutination inhibition (HI), and samples with titers >20 were considered seropositive (*7*). Positive I-ELISA and HI samples were confirmed by using a serum neutralization (SN) assay (*7**,**8*). A sample was considered seropositive when it had an SN titer of >4, determined during experiments on laboratory-injected sheep (*7**–**9*) and testing of wild and domestic African ruminants (*7*). The SN assay has been shown to be highly specific; cross-reactivity with other viruses from the family *Bunyaviridae* is not likely (*9*).

Of the 26 goat samples tested for RVFV, 6 tested positive to RVFV (Table). Samples from 5 goats from the rural villages and 1 from the urban market had RVFV IgG PP >10. Samples from 2 goats from the rural villages had high RVFV HI titers (320 and 5,120). Three of the 6 samples from the rural villages with high IgG PP and HI titers had elevated neutralization titers (>4).

**Table:** Site, age, and sex of goat samples positive for Rift Valley fever virus*

Site	Age, y	Sex	I-ELISA PP	HI titer	SN titer
Market	1.5	F	10	–	/
Rural	3	M	14	–	/
Rural	3.5	F	15	–	/
Rural	4	F	16	–	/
Rural	4.5	M	35	–	4
Rural	3	F	/	320	8
Rural	2	F	106	5,120	12

*By indirect enzyme-linked immunosorbent assay (I-ELISA), hemagglutination inhibition (HI), and serum neutralization (SN); PP, optical density expressed as a percentage of high-positive control serum; /, not tested; –, negative.

The results indicate for the first time that RVFV is present in forests of southern Cameroon. Given the ages of the seropositive goats (2, 3, and 4.5 years), transmission of the virus occurred recently.

In savanna goat herds in northern Cameroon, RVFV IgG prevalence has been reported at 9% to 20% (*2*). To determine prevalence of RVFV in goats in southern Cameroon, more animals need to be sampled; the small sample size and isolation of the few rural villages are unrepresentative.

The presence of RVFV antibodies in domestic animals suggests that this virus may also be circulating in human populations, despite the absence of reports. A study of 21 persons in Ngoïla in southern Cameroon found no RVFV antibodies during a bloody diarrhea epidemic in 1998 (*4**,**10*); however, testing facilities for RVFV are not available in Cameroon, and the general population and healthcare providers have limited awareness of the virus and associated disease.

Central African forests have a high diversity of forest ungulates (>10 species), and RVFV has been reported from a number of wild African ungulates (*7*). The potential for exchange of RVFV and other pathogens between goats and wild ungulates could have substantial effects on animal production and on the conservation of wild species, some of which are considered near-threatened. Livestock disease surveillance can play a role not only in assessing the distribution of livestock pathogens but also in monitoring disease emergence.
